# Intertwined -isms: an exploration of relationships between ageism and sexism in workplace and non-work contexts

**DOI:** 10.3389/fpsyg.2023.1138812

**Published:** 2023-07-17

**Authors:** Sangkyung Bae, Moon Choi

**Affiliations:** Graduate School of Science and Technology Policy, Korea Advanced Institute of Science and Technology (KAIST), Daejeon, Republic of Korea

**Keywords:** discrimination, oppression, diversity, intergenerational relationships, Workplace Intergenerational Climate Scale (WICS), older workers

## Abstract

This study aimed to examine the association of workplace-based ageism with (a) ageism in non-work contexts and (b) workplace-based sexism. Data came from an online survey of workers in South Korea, with a sample stratified by gender and age group (*N* = 600; mean age = 43.6  years, range 20–74). Workplace-based ageism was measured using the Workplace Intergenerational Climate Scale (WICS). Other measures included the Fraboni Ageism Scale (FAS) and the Workplace Sexism Culture Scale (WSCS). A series of logistic regression models for endorsing the most workplace ageism (i.e., scoring in WICS bottom quartile) were estimated. Results showed that with each unit increase in FAS scores, the probability of belonging to the WICS bottom quartile increased by 7% while controlling for sociodemographic characteristics [odds ratio (OR) = 1.07, 95% confidence interval (CI) = 1.04–1.10, *p* < 0.01]. Likewise, when WSCS scores increased by one unit, the probability of belonging to the WICS bottom quartile increased by 8% while controlling for sociodemographic characteristics (OR = 1.08, 95% CI = 1.04–1.12, *p* < 0.01). Findings suggest that ageism and sexism may be intertwined across workplace and non-work contexts.

## 1. Introduction

According to the World Population Prospects (2022) from the United Nations, population aging has been progressing rapidly in South Korea as the proportion of older adults (i.e., aged 65 and over) has increased from 7.1% in 2000 to 16.7% in 2021. Mirroring this rapid population aging, the proportion of workers aged 55 and older in South Korea has almost doubled from 15.2% to 32.8% within the past 20 years (Statistics Korea, 2022). Due to this drastic change in workforce demographics, multiple generations with different experiences and work values have to work together (Bal et al., 2011).

A number of previous studies have reported that ageism in the workplace is severe and prevalent with negative effects on employees’ mental health and overall job satisfaction (Lagacé et al., 2019; Marchiondo et al., 2019). Previous studies have contributed to better understanding of ageism in the workplace, but with notable limitations, as follows. First, the majority of previous studies have explored workplace-based ageism toward a single age group, i.e., older workers (e.g., Lu, 2010). Second, most of them have examined ageism primarily in a single specific context such as the medical field, without exploring its associations across different contexts (e.g., Muangpaisan et al., 2008). Finally, little has been studied about the association of ageism with sexism in the workplace. Previous research on the interrelationships between different forms of demographic-based discrimination has tended to focus on the interplay between sexism and racism (e.g., Stevens-Watkins et al., 2014), while little attention has been given to the association between sexism and ageism despite the rising severity of ageism.

Although ageism is typically perceived as stereotypes, prejudice, and discrimination towards *older* workers, it may also be directed towards workers from other age groups (Marchiondo et al., 2016). For instance, younger workers may face disadvantages in the workplace due to the seniority system, which rewards older individuals with greater authority (Ryu and Cervero, 2011; Xiao et al., 2013). Ageism affects all age groups and plays a dynamic role, regardless of the degree of its impact on different age groups. In light of this, King and Bryant (2017) developed a scale to assess workers’ ageist attitudes and perceptions about co-workers in different age groups, i.e., the “Workplace Intergenerational Climate Scale (WICS).” Considering the increase in diversity of employees’ age composition and the importance of intergenerational relations in the workplace, this validated scale is useful in examining attitudes of age-inclusiveness of both younger and older workers (Stiehr and Vandermause, 2017). Since the WICS was introduced in 2017, a total of 27 journal articles citing this scale have been published (*Web of Science,* January 6, 2023). About two thirds of these (19 out of 27) were published in management or gerontology journals; and many of them focused on understanding factors and consequences associated with age-diversity climate such as feelings of satisfaction at work or work engagement (e.g., Lagacé et al., 2019; Firzly et al., 2021; McConatha et al., 2022).

One of the earliest theories in the literature on prejudice posits that personality plays a role in influencing prejudice (Allport, 1954). According to Allport (1954), prejudicial thinking is not limited to having a negative attitude towards one specific group, but rather, it is a way of thinking about others and the world around one. Adorno et al. (1950) developed the theory of the authoritarian personality which can be used to explain the relationship between personality and prejudice. In addition to these earlier theories, Social Dominance Theory suggests that all types of group-based oppression are special cases of the human tendency to establish hierarchies based on group membership, and that both individual and structural factors contribute to them (Sidanius et al., 2004).

Previous studies have empirically examined the interconnections among various forms of group-based oppression, such as between racism and sexism (e.g., (Sidanius, 1993 Glick and Fiske, 1996) and between sexual prejudice and sexism (e.g.,^3,1009^). However, conspicuously, only few studies have empirically investigated the associations between sexism and ageism. For example, Chonody and Wang (2014) examined the association between ageism and sexism among social work faculty and found no statistically significant association. Also, Aosved et al. (2009) reported that various types of intolerance are distinct and interconnected constructs, and certain constructs exhibit stronger correlations with each other (e.g., sexism and sexual prejudice; sexism and racism) compared to others (e.g., ageism and sexual prejudice). However, the theoretical connection between various forms of oppression indicates a need for further exploration of their associations.

**Figure 1:**
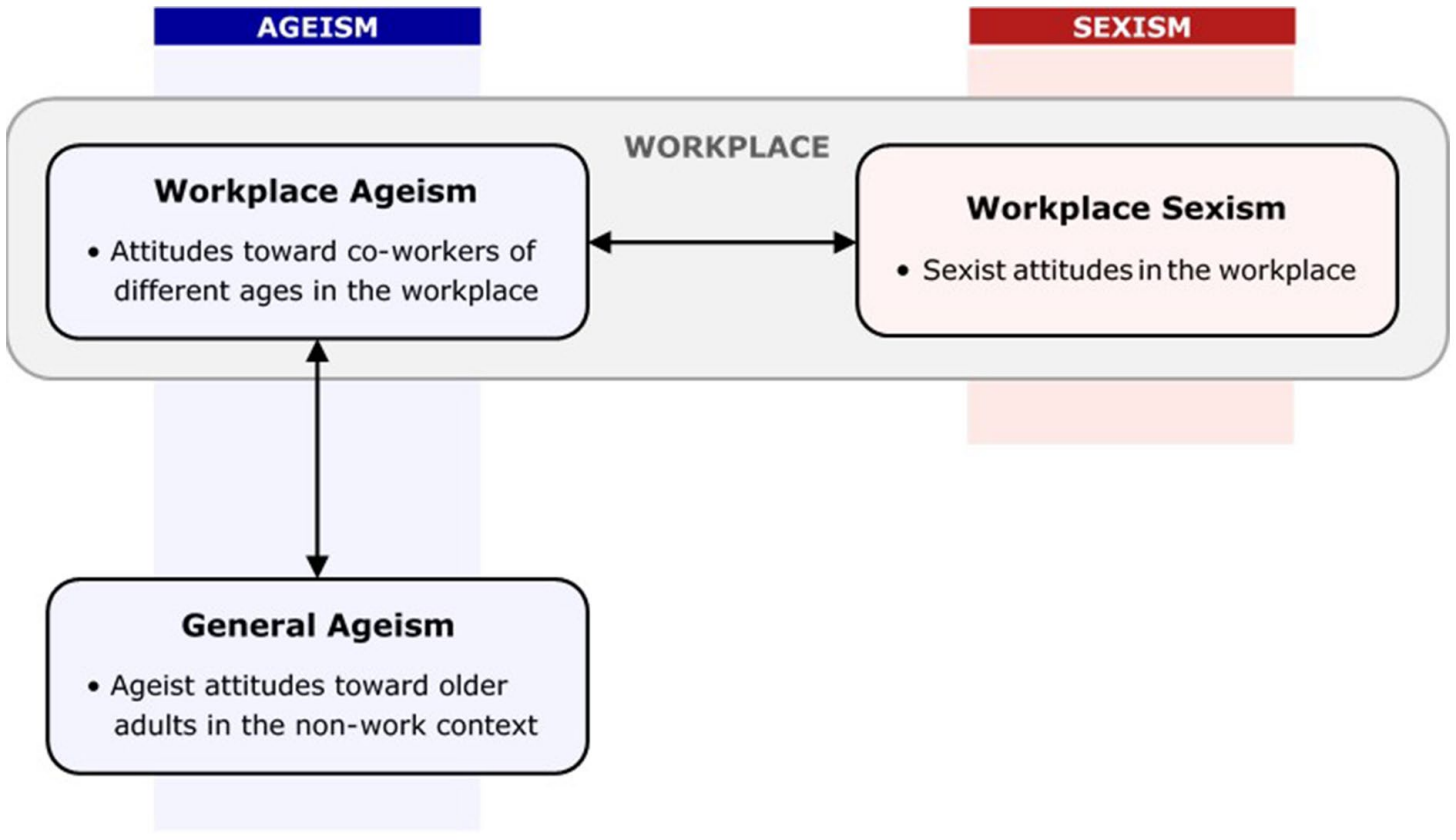
Conceptual model.

Therefore, to fill these knowledge gaps, with a focus on ageism in the workplace, this study aimed to add further understanding by investigating (a) the association between ageism in workplace and non-work contexts and (b) the association between ageism and sexism in the workplace context (see Figure 1). The study presented two hypotheses. The first hypothesis proposed a positive association between ageism in the workplace and outside of work. The second hypothesis suggested that workers with more ageist attitudes would also have higher levels of sexist attitudes.

## 2. Methods

### 2.1. Participants

Data came from an online survey conducted in South Korea in January 2021. Survey participants were recruited through one of the largest online panels in South Korea, with a pool of 1.6 million prospective participants. The online panel survey platform has been designed to prevent bot responses. Eligibility for participation in this study required the following three characteristics: (a) working full-time, (b) working for a company/organization with five or more employees, and (c) working in an environment wherein individuals have to interact with other co-workers on a daily basis. This study employed a stratified sampling procedure based on gender and age groups (*N* = 600; mean age = 43.6 years; standard deviation of age = 11.9; 31.8% women). Participants answered the survey questions on the web or a mobile device and received a compensation payment approximately two US dollars for completion. The Institutional Review Board at KAIST approved this study, and all the participants provided informed consent.

### 2.2. Measures

#### 2.2.1. Workplace ageism

Employees’ attitudes and perceptions about co-workers of different ages in the workplace were measured by the Workplace Intergenerational Climate Scale (WICS) (King and Bryant, 2017). The measure contains 20 items such as “Working with co-workers of different ages enhances the quality of my work life,” and “I enjoy interacting with co-workers of different generations.” The WICS was initially translated from English to Korean by the first author. Subsequently, a professional who is fluent in both English and Korean translated the Korean version of the WICS back into English. Two individuals then compared this translation with the original WICS and made modifications until the bi-directional translations matched the original. Participants were asked to respond to each item using a 4-point Likert scale from 1 = Strongly Disagree to 4 = Strongly Agree. Higher scores mean less discriminatory attitudes toward co-workers in different age groups.

#### 2.2.2. General ageism

Ageist attitudes toward older adults in the non-work context were assessed with the Korean version of Fraboni Ageism Scale (FAS) proposed by Kim et al. (2012), which consists of 18 items (5-point Likert scale from 1 = Strongly Disagree to 5 = Strongly Agree). Higher scores reflect more discriminatory attitudes.

#### 2.2.3. Workplace sexism

A culture of sexist attitudes in the workplace was measured with the Workplace Sexism Culture Scale (WSCS), developed by Ahn et al. (2010), consisting of 10 items (5-point Likert scale from 1 = Strongly Disagree to 5 = Strongly Agree). Higher scores reflect more sexist attitudes. The complete list of questionnaire items is available in the supplementary materials.

### 2.3. Analytic strategy

We aimed to examine the associations of workplace ageism (WICS scores) with both ageism in non-work contexts (FAS scores) and workplace sexism (WSCS scores), rather than explore the implications of WICS scores or find a cut-off value of WICS. Therefore, we grouped study participants by their WICS scores: the lowest quartile vs. the others. This yielded a sufficient number of participants to estimate analytic model fit of associations with workplace ageism (Boyd et al., 2005). First, bivariate analyses were conducted comparing the two groups (bottom 25% vs. other 75%) in terms of their sociodemographic characteristics, general ageism in the non-work context, and workplace sexism, using chi-square tests for categorical variables and independent-sample *t* tests for continuous variables. Next, a series of logistic regression models for the WICS bottom quartile group were estimated, using IBM SPSS V. 25.0 statistical software. All *p* values refer to two-tailed tests; and the alpha level was set equal to 0.05 for all statistical analyses.

## 3. Results

Participants in the WICS bottom quartile, those with the most workplace ageism, had significantly higher scores on the Fraboni Ageism Scale (FAS) as well as the Workplace Sexism Culture Scale (WSCS), compared to those in the other 75% (44.6 vs. 41.4 and 24.1 vs. 22.0, respectively) (see Table 1). With regard to sociodemographic characteristics, only marital status was significantly different between the two groups; those in the WICS bottom quartile were less likely to be married and living with a spouse compared to the others (48.9% vs. 60.0%).

**Table 1:** Bivariate analysis results: WICS bottom 25% vs. other 75%.

	Total (*N* = 600)	Bottom 25% (*n* = 182)	Others 75% (*n* = 418)	Chi-square test or *t*-test	*p*-value
Sociodemographic characteristics					
Age, *M (SD)*	43.6 (11.9)	43.0 (11.3)	43.7 (12.0)	−0.66	0.50
Woman, % (*n*)	31.8% (191)	33.0% (60)	31.3% (131)	0.15	0.69
Married and living with spouse, % (*n*)	**56.7% (340)**	**48.9% (89)**	**60.0% (251)**	**6.41**	**0.01** ^*^
Education—college or above, % (*n*)	92.0% (552)	92.3% (168)	91.9% (384)	0.03	0.85
Scales					
Fraboni Ageism Scale (Korean version), *M (SD)*	**42.3 (7.2)**	**44.6 (5.9)**	**41.4 (7.5)**	**5.11**	**0.00** ^**^
Workplace Sexism Culture Scale, *M (SD)*	**22.6 (5.5)**	**24.1 (5.0)**	**22.0 (5.6)**	**4.70**	**0.00** ^**^

WICS, Workplace Intergenerational Climate Scale; Bottom 25% = Participants who scored in the lowest quartile of the WICS; Other 75% = The remaining participants who scored higher than the bottom 25% of the WICS; statistically significant results (*p* < 0.05) are shown in boldface. ^*^p < 0.05; ^**^p < 0.01.

The logistic regression estimates showed that those with more ageist attitudes toward older adults in the non-work context were more likely to belong to the WICS bottom quartile, as predicted in the first hypothesis. Specifically, with one unit increase in the FAS scores, the probability of belonging to the WICS bottom quartile increased by 7% while controlling for sociodemographic characteristics [odds ratio (OR) = 1.07; 95% confidence interval (CI) = 1.04–1.10; *p* < 0.01]. Likewise, consistent with the second hypothesis, endorsing more workplace sexism was associated with more workplace ageism. With one unit increase in the WSCS scores, the probability of belonging to the WICS bottom quartile increased by 8% while controlling for sociodemographic characteristics (OR = 1.08; 95% CI = 1.04–1.12; *p* < 0.01).

The final logistic regression model included both the FAS and the WSCS in addition to sociodemographic characteristics. This model showed that even after controlling for other types of discriminatory attitudes, general ageism in the non-work context and workplace sexism were still significantly associated with workplace ageism (OR = 1.05, 95% CI = 1.02–1.08 and OR = 1.04, 95% CI = 1.00–1.09), respectively. In addition, those who were married and living with their spouse were less likely to belong to the WICS bottom quartile compared to those with another marital status (OR = 0.53, 95% CI = 0.33–0.83, *p* < 0.01) (see Table 2).

**Table 2:** Results of logistic regression models for the WICS bottom quartile (*N* = 600).

Variables	Model 1		Model 2		Model 3		Model 4		Model 5	
	OR	95% CI	OR	95% CI	OR	95% CI	OR	95% CI	OR	95% CI
Fraboni Ageism Scale (Korean version)	**1.07** ^**^	**1.04–1.10**	**1.07** ^**^	**1.04–1.10**	–	–	–	–	**1.05** ^**^	**1.02–1.08**
Workplace Sexism Culture Scale	–	–	–	–	**1.08** ^**^	**1.04–1.11**	**1.08** ^**^	**1.04–1.12**	**1.04** ^*^	**1.00–1.09**
Age	–	–	1.02	1.00–1.04	–	–	1.02	1.00–1.04	1.02	1.00–1.04
Gender	–	–	1.07	0.70–1.66	–	–	0.94	0.61–1.44	1.03	0.66–1.59
Education—college or above	–	–	1.06	0.54–2.09	–	–	1.15	0.59–2.25	1.07	0.54–2.12
Married and living with spouse	–	–	**0.54** ^**^	**0.34–0.85**	–	–	**0.53** ^**^	**0.33–0.83**	**0.53** ^**^	**0.33–0.83**
NagelKerke *R^2^*	0.06		0.08		0.05		0.06		0.09	

WICS bottom quartile = Participants who scored in the lowest quartile in WICS; OR, Odds Ratio; 95% CI, 95% Confidence Interval; statistically significant results (*p* < 0.05) are shown in boldface. ^*^p < 0.05; ^**^p < 0.01.

## 4. Discussion

The primary findings from this study are that discriminatory attitudes toward different age and gender groups in both workplace and non-work contexts are intertwined. Individuals with more ageist attitudes towards co-workers in different age groups in the workplace were more likely to have ageist attitudes towards older adults in non-work contexts, and they were also more likely to have sexist attitudes in the workplace. These findings suggest that individuals with certain discriminatory attitudes in one context tend to hold similar discriminatory attitudes in other contexts, which is consistent with Allport’s (1954) concept of prejudice-proneness, i.e., that the prejudiced person in one domain is more likely to have prejudices in other domains as well. Additionally, this finding can be interpreted as evidence of the effect of an individual’s personality traits, such as openness to experience and agreeableness as described by the Five Factor theory, on negative attitudes towards out-groups (Sibley and Duckitt, 2008). Previous studies have suggested that an individual’s low openness to experience and agreeableness are associated with prejudice-proneness and posit the existence of an Authoritarian Personality Theory. This theory is associated with a Social Dominance Orientation and Right-Wing Authoritarianism which may mediate the relationship between personality traits and discriminatory attitudes (Jackson and Poulsen, 2005 Sibley and Duckitt, 2008). In other words, an individual’s lack of openness to other out-groups, which leads to having more demographic-based discriminatory attitudes in the domain of demographic factors, may be associated with different kinds of discriminatory attitudes.

Marital status was the only sociodemographic characteristic associated with ageist attitudes toward co-workers in different age groups in the workplace. This study did not measure self-esteem, but previous studies have suggested that people with high explicit but low implicit self-esteem may be more likely to discriminate based on ethnicity (e.g., Jordan et al., 2005). Additionally, relationship status has been reported to be associated with self-esteem (Grundström et al., 2021). Building on these previous studies, self-esteem could potentially mediate the relationship between marital status and ageism in the workplace. Additionally, marital status could affect daily contacts with different age groups, such as spouse’s relatives, due to increased expectations for family obligations. These increasing contacts may be associated with better understanding of people in other age groups (e.g., Allport, 1954; Levy, 2018). Future research needs to investigate these potential mechanisms of the relationship between marital status and workplace ageism further.

Study findings should be interpreted in light of limitations. First, the data were obtained from individuals who voluntarily participated in an online survey. Accordingly, self-selection bias cannot be ruled out, as only those who were willing to provide their information through online platforms participated (Bethlehem, 2010). Second, the sample was drawn from one county, i.e., South Korea. Therefore, the findings need be interpreted considering the socio-cultural context, wherein the seniority system is often common in workplace contexts. In a seniority-based system, job tenures secured by the accumulated working years are one of dominant factors beyond competence and performance for determining basic salaries and compensation (Horak and Yang, 2019). Third, an individual’s psychological characteristics, such as personal traits and self-esteem, which have a potential mediating effect on discriminatory attitudes (Jordan et al., 2005; Grundström et al., 2021), were not considered in this study. Despite these limitations, study findings contribute to understanding intertwined ageism and sexism in the workplace as well as ageism across workplace and non-work contexts.

Strengths of this study include several novel aspects to the research. First, this study has focused on current full-time workers and their attitudes toward co-workers in different age groups. Second, most previous studies focused on ageism only toward older workers or older adults; however, this study has contributed to understanding discriminatory attitudes toward different age groups, recognizing that ageism affects both young and older people (Marchiondo et al., 2016). Furthermore, to our knowledge, this study is the first one using the Workplace Intergenerational Climate Scale (WICS) in South Korea. Third, unlike most previous studies focused on one context, this study has contributed to understanding the associations of ageism between workplace and non-work contexts. Finally, this study is one of very few exploring the associations between ageism and sexism in the workplace.

This study’s findings indicate that interventions to reduce ageism in the workplace may have positive impacts on alleviating other types of discriminatory attitudes in other contexts. To this end, future research should further investigate the association between various forms of oppression in workplace and non-work contexts, including the association between workplace ageism and sexism in non-work contexts, which was not included in the scope of this study. Additionally, future research should investigate the fundamental mechanisms of the systematic nature of discriminatory attitudes. Based on this understanding, researchers can develop effective evidence-based interventions to alleviate various types of discriminatory attitudes.

## Data availability statement

The original contributions presented in the study are included in the article/Supplementary material, further inquiries can be directed to the corresponding author.

## Ethics statement

The studies involving human participants were reviewed and approved by KAIST Institutional Review Board. The patients/participants provided their written informed consent to participate in this study.

## Author contributions

SB and MC contributed to the conception and design of the study and wrote the first draft of the manuscript. SB organized the database and performed the statistical analysis. All authors contributed to the article and approved the submitted version.

## Funding

This work was supported by Institute for Information & Communications Technology Planning & Evaluation (IITP) grant funded by the Korean government (MSIT) (No. 2019-0-01396, Development of framework for analyzing, detecting, mitigating of bias in AI model and training data) as well as the KAIST Basic Faculty Research Fund.

## Conflict of interest

The authors declare that the research was conducted in the absence of any commercial or financial relationships that could be construed as a potential conflict of interest.

## Publisher’s note

All claims expressed in this article are solely those of the authors and do not necessarily represent those of their affiliated organizations, or those of the publisher, the editors and the reviewers. Any product that may be evaluated in this article, or claim that may be made by its manufacturer, is not guaranteed or endorsed by the publisher.
