# Supplementary material for: Intertwined -isms: an exploration of relationships between ageism and sexism in workplace and non-work contexts
Source: Front Psychol. 2023 Jul 17;14:1138812. doi: 10.3389/fpsyg.2023.1138812 (PMC10389284; doi:10.3389/fpsyg.2023.1138812)
Supplement: Supplementary file 1 [file Data_Sheet_1.docx]

**Appendix 1**

*Workplace Intergenerational Climate Scale (WICS) (Workplace Ageism)*

| **Lack of Generational Stereotypes (LGS)** |
| --- |
| Co-workers outside my generation are not interested in making friends outside their generation. (r)  Co-workers outside my generation complain more than co-workers my age do. (r)  Co-workers outside my generation usually talk about things that don’t interest me. (r)  Co-workers outside my generation tend to work differently than co-workers my age do. (r) |
| **Positive Intergenerational Affect (PIA)** |
| I feel comfortable when co-workers outside my generation try to make conversation with me.  I enjoy interacting with co-workers of different generations.  My co-workers outside my generation are interesting and unique individuals.  People work best when they work with others their same age. (r) |
| **Intergenerational Contact (IC)** |
| How often do you have conversations with co-workers outside your generation?  How often do you have conversations with co-workers outside your generation relating to things other than work?  How often do you talk with co-workers outside your generation about your personal lives?  How often do you eat meals with co-workers outside your generation during the workday? |
| **Workplace Generational Inclusiveness (WGI)** |
| I believe that my work environment is a healthy one for people of all ages.  Workers of all ages are respected in my workplace.  I am able to communicate effectively with workers of different generations.  Working with co-workers of different ages enhances the quality of my work life. |
| **Workplace Intergenerational Retention (WIR)** |
| My co-workers make older workers feel they should retire. (r)  I feel pressure from younger workers to step down. (r)  I feel pressure from older workers to step down. (r)  In my workplace, qualified younger workers tend to be overlooked for promotions. (r) |

*Note*. (r) = reverse scored.

**Citation:** King, S. P., & Bryant, F. B. (2017). The Workplace Intergenerational Climate Scale (WICS): A self‐report instrument measuring ageism in the workplace. *Journal of Organizational Behavior, 38*(1), 124-151.

**Appendix 2**

*The Korean Version of Fraboni Ageism Scale (FAS) (General Ageism)*

| **Affective avoidance** |
| --- |
| I personally would not want to spend much time with an older person.  I would prefer not to go to an open house at a seniors’ club, if invited.  I would prefer not to live with an older person.  The company of most older people is quite enjoyable. (r)  I don’t like it when older people try to make a conversation with me.  Most older people are interesting, individualistic people. (r)  I sometimes avoid eye contact with older people when I see them. |
| **Discrimination** |
| It is best that older people live where they won’t bother anyone.  Most older people should not be allowed to renew their driver’s licenses.  Older people don’t really need to use our community sports facilities.  Older people deserve the same rights and freedoms as do other members of the society. (r)  It is sad to hear about the plight of older people in our society these days. (r) |
| **Stereotype** |
| Many older people just live in the past.  Feeling depressed when around older people is probably a common feeling.  Older people complain more than other people do.  Many older people are not interested in making new friends and prefer to be in the circle of friends they have had for years.  Many older people are happiest when they are with people in their own age.  Many older people are stingy and hoard their money and possessions. |

*Note*. (r) = reverse scored.

**Citation:** Kim, J. Y., Kim, M. H., & Min, K. H. (2012). *Han-guk-pan no-in-cha-byeol-ju-ui cheok-do-ui ta-dang-hwa: Dae-hak-saeng pyo-bon-eul dae-sang-eu-ro* [Validation of the Korean version of the Fraboni Ageism Scale (FSA): A study of Korean university students]. *Korean Journal of Social and Personality Psychology, 26*(4), 89-106.

**Appendix 3**

*Workplace Sexism Culture Scale (WSCS) (Workplace Sexism)*

| In my workplace important information is transmitted through a private network of men. (r)  In my workplace, work for women and men is substantially divided. (r)  My workplace applies, not officially but unofficially, different personnel evaluation standards to men and women.  The CEO of my workplace treats every man and woman equally. (r)  In my workplace, female employees mostly resign before or after pregnancy or childbirth.  In my workplace, the opportunities for education or training to produce key leaders mostly go to male employees.  In my workplace, female employees tend to be evaluated based on their looks or sexual attractiveness.  In my workplace, it is not comfortable to leave the office on time.  Working late into the night is usually seen as loyalty to the organization or a sign of diligence.  People often complain about female employees because it is not easy to ask them to work overtime or at night. |
| --- |

*Note*. (r) = reverse scored.

**Citation:** Ahn, S. S., Park, S. J., & Kim, K. M. (2010). *Sung-pyeong-deung sil-cheon kook-min sil-tae-jo-sa mit jang-ae-yo-in-yeon-gu* (Ⅱ) [Public survey on the practices of gender equality and its barriers: Focusing on activities in the workplace (Ⅱ)] (No. 2010-6). Korea Women’s Development Institute.
